# Insights Into Low‐Temperature Cation Ordering in Fe‐Added Ce–Zr‐Based Oxides

**DOI:** 10.1002/smll.202412830

**Published:** 2025-03-23

**Authors:** Yume Okazaki, Akihiro Ishii, Itaru Oikawa, Hitoshi Takamura

**Affiliations:** ^1^ Department of Materials Science Graduate School of Engineering Tohoku University 6‐6‐02 Aramaki Aoba Sendai 980–8579 Japan

**Keywords:** DFT calculations, Fe dissolution, high‐temperature in situ XRD, low‐temperature cation ordering, oxygen partial pressure, oxygen storage capacity, Zr‐rich Ce–Zr

## Abstract

CeO_2_–ZrO_2_ (CZ) solid solutions are widely utilized to control the oxygen partial pressure of automobile exhaust purification systems owing to their high oxygen storage capacity (OSC) related to the valence change of Ce ions upon reduction. Among various CZs, cation‐ordered κ‐Ce_2_Zr_2_O_8_ shows the highest OSC; however, the ordering requires high‐temperature reduction above 1200 °C, causing grain growth and potentially compromising the OSC. Recently, it has been reported that adding a small amount of Fe_2_O_3_ to CZ (Zr/Ce = 1) lowers the ordering temperature to 800 °C. In this study, Zr‐rich CZ, known for its excellent heat resistance and widespread applications, is cation‐ordered at low temperatures by the addition of Fe_2_O_3_. Using high‐temperature in situ XRD, the low‐temperature ordering behavior of Fe_2_O_3_‐added Zr‐rich CZ is observed under oxygen partial pressure during reduction. A weakly reducing atmosphere promotes CZ ordering because Fe_2_O_3_ remains an ionic Fe that can be dissolved in CZ to facilitate cation migration. In contrast, a strongly reducing atmosphere converts Fe_2_O_3_ to metallic Fe, which is unfavorable for CZ ordering. The study suggests that the reduction atmosphere has a significant impact on the dissolution of transition metal oxides and cation ordering of ceramics.

## Graphical abstract

Cation ordering of Fe_2_O_3_‐added Zr‐rich CZ is achieved at 800 °C under a weakly reducing atmosphere because Fe_2_O_3_ is reduced to FeO dissolved in CZ to facilitate cation migration. Conversely, in a strongly reducing atmosphere, Fe_2_O_3_ is reduced to metallic Fe, which cannot contribute to ordering. Thus, controlling the oxygen partial pressure is important to promote the ordering of CZ.

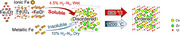

## 1 Introduction

Reversible redox reactions are critical in many energy systems, such as fuel cells, water electrolysis devices, all‐solid‐state batteries, and catalysts, as they determine the performance and durability of these systems. For example, the durability of solid‐oxide electrolysis cells (SOECs) is governed by the redox reactions of Ni‐based composite cathodes, which cause severe Ni migration and dissipation.^[^ ^1^ ^]^ This study focuses on the reversible redox reactions in oxygen storage materials used in exhaust gas purification catalysts. Despite the rapid growth of electric vehicles (EVs), combustion engines and hybrid vehicles are expected to remain in use, as power shortages associated with EV recharging cannot be eliminated in the foreseeable future. To comply with stringent automobile exhaust emission regulations, it is imperative to improve the performance of gas purification systems and use three‐way catalysts to remove carbon monoxide, hydrocarbons, and nitrogen oxides.^[^ ^2^, ^3^ ^]^ Incorporating oxygen storage materials into three‐way catalysts allows for stable purification reactions even in environments where the exhaust gas components change within the order of milliseconds.^[^ ^4^, ^5^, ^6^ ^]^

CeO_2_–ZrO_2_ (CZ) is widely used as an oxygen storage material with a high oxygen storage capacity (OSC).^[^ ^7^, ^8^, ^9^, ^10^ ^]^ The valence state of Ce changes from Ce^4+^ to Ce^3+^ to satisfy the electroneutrality condition in Equation (1), leading to an oxygen‐release reaction, as shown in Equation (2).^[^ ^11^, ^12^, ^13^, ^14^, ^15^ ^]^

(1)

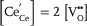

(2) $$\;{\mathrm{CeO}}_{2}\to {\mathrm{CeO}}_{2-\delta }+\frac{\delta }{2}O_{2}$$

Theoretically, a higher Ce content should result in a larger OSC; however, in practice, the proportion of Ce contributing to the OSC is greater on the Zr‐rich side (Zr/Ce > 1) because of the unfavorable increase in elastic energy owing to reductive expansion.^[^ ^16^ ^]^ Thus, Zr‐rich compositions are practically used and exhibit excellent heat resistance.^[^ ^17^, ^18^, ^19^, ^20^, ^21^ ^]^

Another method for increasing the OSC of CZ is cation ordering. For example, t′‐Ce_0.5_Zr_0.5_O_2_, in which Ce and Zr are randomly arranged at a Zr/Ce ratio of 1, has a Ce reduction ratio of only 51.7% of the theoretical value at 500 °C and a corresponding OSC of 440 µmol‐O_2_ g^−1^.^[^ ^22^ ^]^ In contrast, in the cubic pyrochlore‐like (Ce_2_Zr_2_O_7_) structure, known as the κ phase, the Ce reduction rate reaches 88.6% of the theoretical value, and its OSC can reach 750 µmol‐O_2_ g^−1^.^[^ ^22^ ^]^ This is because the Ce and Zr in the κ phase are periodically aligned in the <110> direction, and the oxygen vacancies formed during reduction are also ordered.^[^ ^23^, ^24^, ^25^ ^]^ However, the preparation of the κ phase requires high‐temperature heat treatments at 1200 °C to significantly reduce t′‐Ce_0.5_Zr_0.5_O_2_,^[^ ^26^ ^]^ which leads to a substantial decrease in the specific surface area. In fact, the specific surface area has been reported to drop to less than 1 m^2^ g^−1^ after cation ordering.^[^ ^27^ ^]^ In addition, the grain growth significantly reduces the OSC in the low‐temperature region (≈400 °C), where the diffusion rate is limited. Therefore, it is necessary to lower the heat treatment temperature for cation ordering. In fact, Ce_0.5_Zr_0.4_Ti_0.1_O_2_ prepared by combustion synthesis at 400 °C exhibited an excellent OSC of 655 µmol‐O_2_ g^−1^, even at a low temperature of 200 °C.^[^ ^28^ ^]^ The OSC improvement is attributed to 1) the increased specific surface area during the combustion synthesis and 2) the charge transfer from oxygen atoms to Ce atoms achieved by Ti substitution.^[^ ^28^, ^29^ ^]^

As a novel technique to prepare a cation‐ordered CZ at lower temperatures, recently, our group proposed that by simply mixing Ce_0.5_Zr_0.5_O_2_ (Zr/Ce = 1) and Fe_2_O_3_ powder, the phase transition temperature to pyrochlore Ce_2_Zr_2_O_7_ was reduced by 400 °C, that is, from 1200 to 800 °C.^[^ ^30^ ^]^ However, the detailed mechanism, particularly the role and existence of iron oxide, remains unknown. In this study, the role of Fe_2_O_3_ in the CZ system with respect to the cation‐ordering behavior was clarified, and the explored system was extended to a commercially used Zr‐rich CZ to prove the effectiveness of simple Fe_2_O_3_ addition. For these purposes, the energetics related to cation ordering in CZ with respect to the Zr/Ce ratio and dissolution energy of the additives are discussed based on first‐principles calculations. The cation‐ordering behavior of Zr‐rich CZ was investigated by high‐temperature in situ XRD with precise partial oxygen pressure, p(O_2_), and control. We found that a weakly reducing atmosphere was more effective than a strongly reducing atmosphere for deep reduction and low‐temperature cation ordering in the Fe_2_O_3_‐added Zr‐rich CZ. Based on these results, a possible mechanism for the low‐temperature cation ordering of Zr‐rich CZ upon the addition of Fe_2_O_3_ is proposed.

## 2 Results and Discussion

### 2.1 DFT Calculation

#### 2.1.1 The Energetics of Cation Ordering in CZ

**Figure 1:**
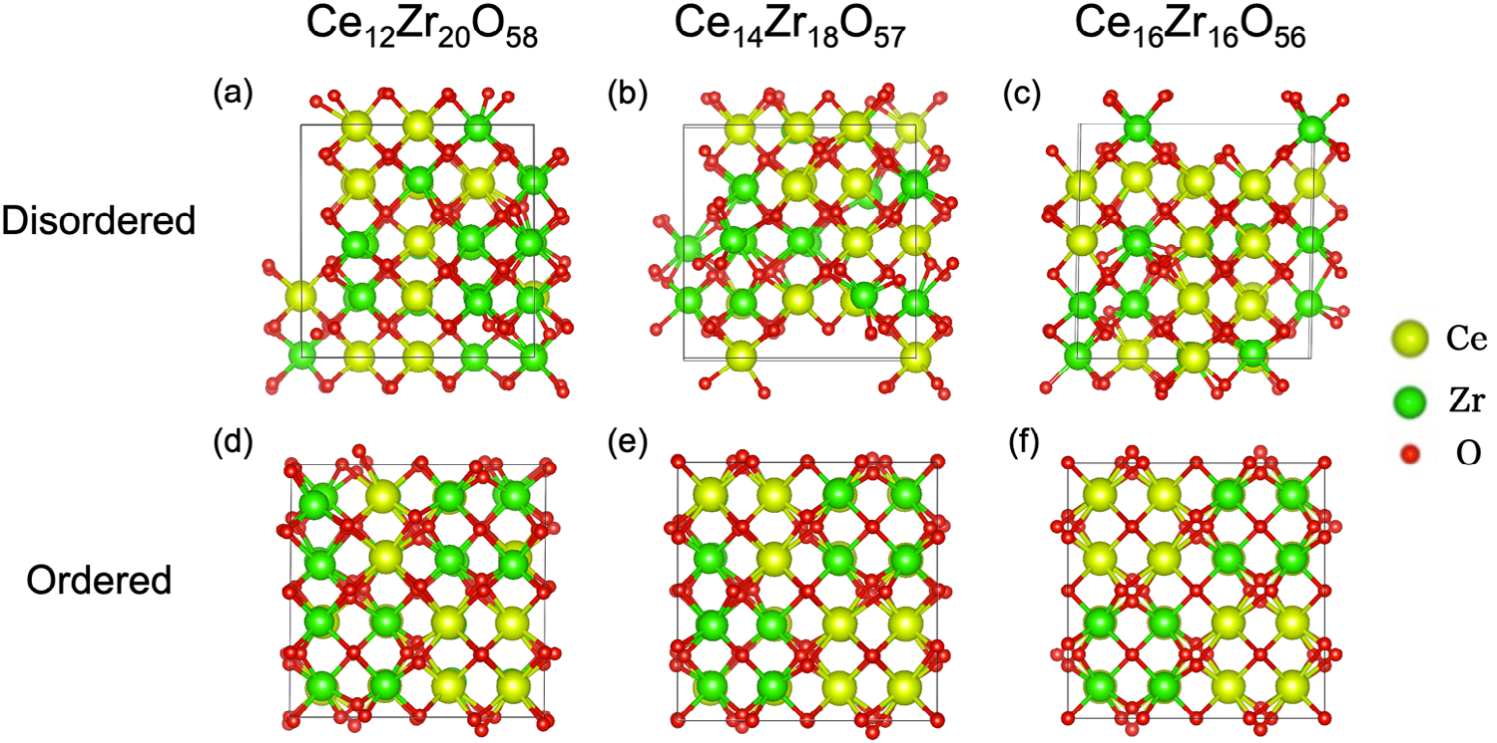
Structural models of disordered a) Zr‐rich Ce_12_Zr_20_O_58_, b) Zr‐rich Ce_14_Zr_18_O_57_, and c) stoichiometric Ce_16_Zr_16_O_56_ (Zr/Ce = 1) and their ordered counterparts d–f), respectively. These correspond to the reduced states (Ce is 3+), and (b) Ce_14_Zr_18_O_57_ is considered a model of the experimentally synthesized Zr‐rich CZ.

At low temperatures, cation‐ordered phases are energetically favored over disordered phases. The energy gain associated with cation ordering was calculated for CZs with different Zr/Ce ratios. To see the effect of Zr‐rich composition clearly, the model of Ce_14_Zr_18_O_57_ (Zr/Ce = 1.29) is selected because the actual Zr‐rich sample is Ce_0.434_Zr_0.566_O_2_ (Zr/Ce = 1.30) based on ICP‐MS. Further Zr‐enriched model, Ce_12_Zr_20_O_58_ (Zr/Ce = 1.67) is also calculated for comparison. Stoichiometric Ce_16_Zr_16_O_56_ (Zr/Ce = 1), representing CZ55 pyrochlore, was also selected as a model case. These compositions correspond to eight times the chemical formula of a typical pyrochlore (A_2_B_2_O_7_). Two types of crystal structure models (disordered and ordered) were prepared for each composition, as shown in **Figure** **1**, where a special quasi‐random structure (SQS) was utilized to prepare the disordered models Figure 1a–c,^[^ ^31^, ^32^ ^]^ and an ordered CZ55 structure Figure 1f was obtained from the CIF database.^[^ ^33^ ^]^ Ordered Zr‐rich CZ structures Figure 1d,e were prepared based on the ordered CZ55 structure (Figure 1f) and the SQS method to substitute excess Zr for Ce sites. These models correspond to the reduced states in which the formal valence of Ce is 3+. Figure 1a–f shows the structures after the optimization of volume and fraction position. **Table** **1** summarizes the total energy of the disordered (*E_dis_*) and ordered (*E_ord_*) models and the energy gain (*ΔE*) associated with cation ordering, that is, the phase transition from the disordered to the ordered phase in the pyrochlore‐type structures. The energy differences were divided by eight to obtain the value per formula unit (A_2_B_2_O_7_). The closer the composition approaches Zr/Ce = 1, the greater the energy gain for order; conversely, the higher the Zr ratio, the smaller the energy gain. In other words, the Zr‐rich CZ was less likely to be ordered than CZ55.

**Table 1:** The total energy of the disordered (*E_dis_*) and ordered (*E_ord_*) phases in the pyrochlore‐type structures of Zr‐rich Ce_12_Zr_20_O_58_, Ce_14_Zr_18_O_57_, and stoichiometric Ce_16_Zr_16_O, and their energy difference (*ΔE = E_ord_*–*E_dis_*). The corresponding local distortion parameters (*Δ*) of the ordered structures are also included (a larger *Δ* indicates greater distortion).

	Ce_12_Zr_20_O_58_	Ce_14_Zr_18_O_57_	Ce_16_Zr_16_O_56_
*E_dis_* (eV)	−803.47	−785.83	−767.82
*E_ord_* (eV)	−808.69	−792.38	−775.84
*ΔE* (eV)	−0.65	−0.82	−1.00
*Δ* (Å^2^)	0.1742	0.0823	0.0000

The differences in the local structures of Zr‐rich CZ and CZ55 were further investigated with respect to cubic symmetry. Cubic structures should have four threefold rotation axes in the direction of the body diagonals, that is [111], $\left[\bar{1}\bar{1}1\right]$, $\left[1\bar{1}\bar{1}\right]$, and $\left[\bar{1}1\bar{1}\right]$. In other words, the atomic positions of identical elements in cubic structures should be invariant with respect to the following symmetry operations:

(3)

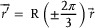

where $\vec{r}$ and $\vec{r^{\prime }}$ are the original and the rotated atomic positions, and $R(\pm \frac{2\pi }{3})$ represents the matrix of three‐fold rotation operations to convert the atomic positions. By summing up the position differences and dividing by the number of atoms (*N* = 90, 89, 88 for Figure 1(a), (b) and (c), respectively), that is,

(4) $$\Delta =\frac{1}{N}\sum_{crystal}{\left(\vec{r^{\prime }}-\vec{r}\right)}^{2}$$

the Δ value can be a good measure of local distortion in cubic crystals. Δ for ordered models, Figure 1d,e,f are also shown in Table 1. The ordered CZ55 (f) preserved the cubic symmetry, whereas the Zr‐rich CZ (d) and (e) showed larger deviations, suggesting a more distorted local environment in the Zr‐rich CZ.

**Figure 2:**
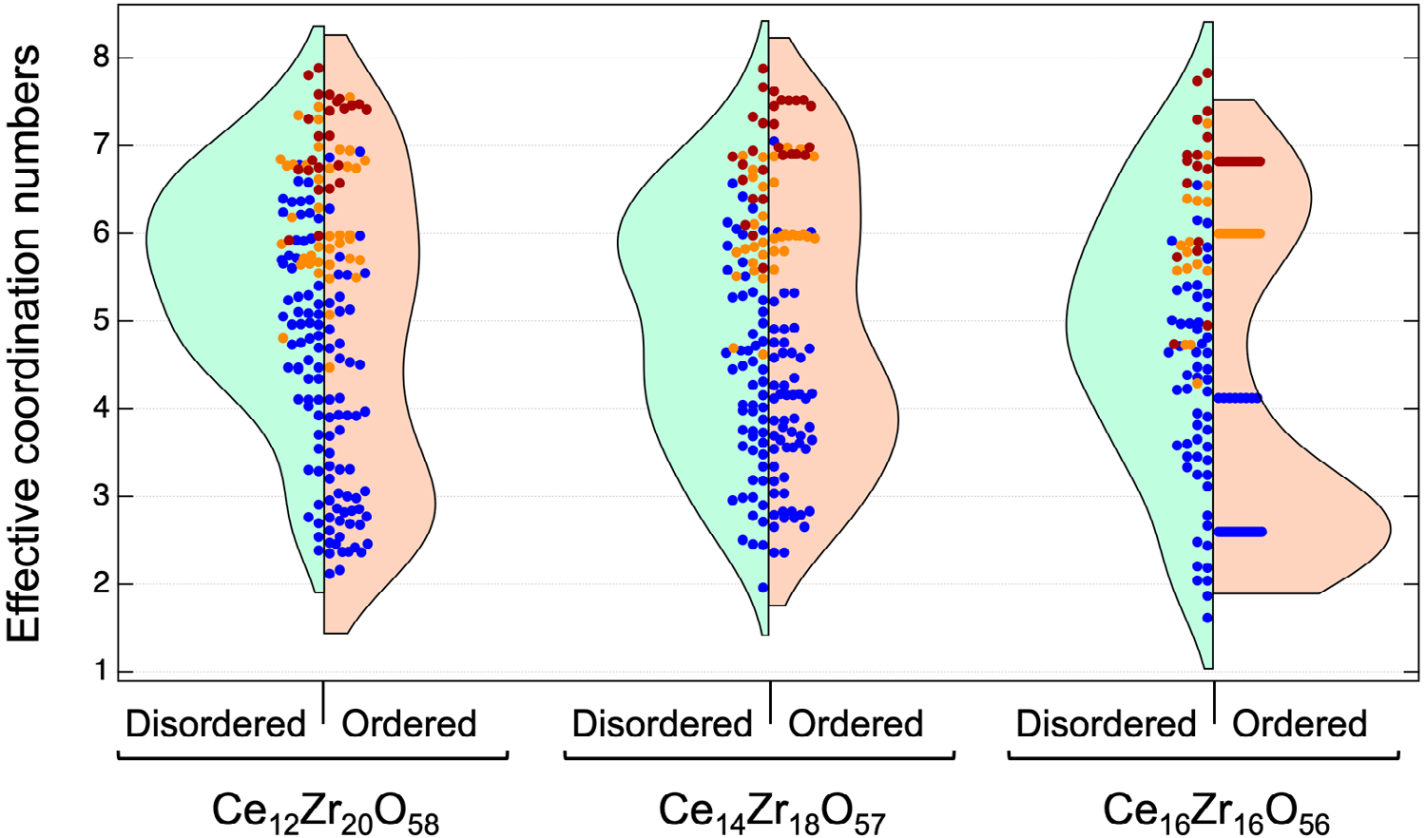
Violin plots of effective coordination numbers (ECNs) of Zr‐rich CZ (Ce_12_Zr_20_O_58_, Zr/Ce = 1.67; Ce_14_Zr_18_O_57_, Zr/Ce = 1.29) and CZ55 (Ce_16_Zr_16_O_56_, Zr/Ce = 1) for ordered and disordered states. ECNs were calculated for the optimized models shown in Figure 1. The ECNs around Ce, Zr, and O are plotted as filled brown, yellow, and blue circles, respectively.

To understand the limited energy gain associated with the cation ordering in Zr‐rich CZs, *ΔE* in Table 1, effective coordination numbers (ECN) were calculated, taking the bond lengths into consideration.^[^ ^34^ ^]^ **Figure** **2** shows the violin plots of the ECNs of Zr‐rich CZ (Ce_12_Zr_20_O_58_, Zr/Ce = 1.67; Ce_14_Zr_18_O_57_, Zr/Ce = 1.29) and CZ55 (Ce_16_Zr_16_O_56_, Zr/Ce = 1) for the ordered and disordered states. The ECNs around Ce, Zr, and O are shown as brown, yellow, and blue‐filled circles, respectively. The disordered states of Zr‐rich CZ and CZ55 and their ordered states shown in Figure 2 correspond to the optimized structures in Figure 1. In the disordered phases, the CZ55 and Zr‐rich CZs are randomly distributed. Meanwhile, upon ordering, the ECNs of CZ55 approach their theoretical values, that is, eight for Ce, six for Zr, and four for O. In contrast, the ECNs in Zr‐rich CZs exhibit a broader distribution than those in CZ55, suggesting that the pyrochlore structure in Zr‐rich compositions (Ce_12_Zr_20_O_58_ and Ce_14_Zr_18_O_57_) is locally less ordered, even when Ce and Zr are ordered. This is consistent with the calculation results, which show that Zr‐rich CZ has a smaller energy associated with ordering compared with CZ55.

#### 2.1.2 The Dissolution Energy of Fe, Sc, and Al into CZ55

**Figure 3:**
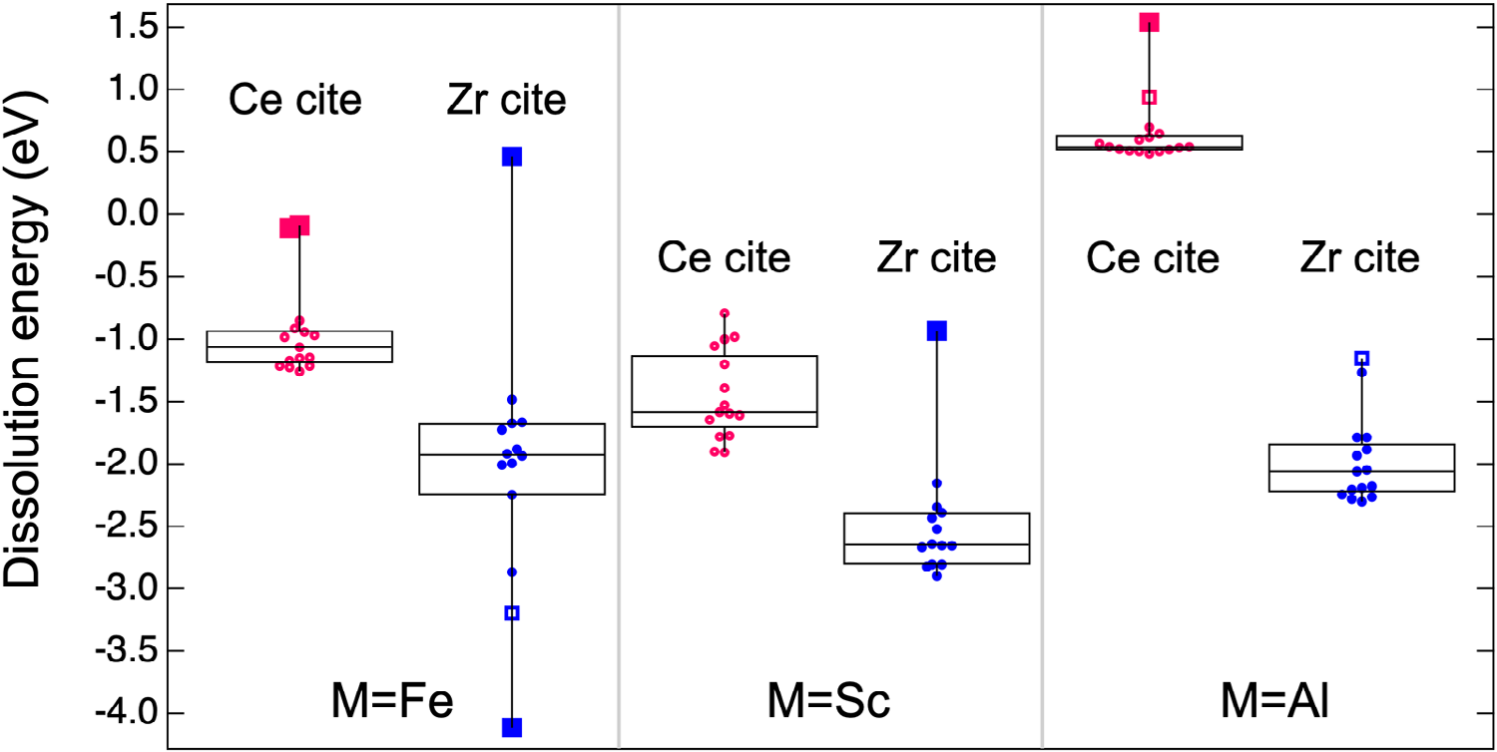
Box plots of the calculated dissolution energies of Fe^3+^, Sc^3+^, and Al^3+^ at the Ce (red) or Zr (blue) sites of Ce_16_Zr_16_O_56_. The median values were used as the dissolution energies for comparison.

After the CZ55 (Zr/Cr = 1) sample was mixed with Fe_2_O_3_ powder, the ordering temperature of the pyrochlore‐type Ce_2_Zr_2_O_7_ was reduced to 800 °C.^[^ ^30^ ^]^ To investigate whether the dissolution of Fe^3+^ ions in CZ55 could be a contributing factor to the low ordering temperature, the dissolution energy of Fe^3+^ ions in ordered CZ55 (Ce_16_Zr_16_O_56_) was calculated using Equations (5) and (6). The dissolution energies of other trivalent ions such as Sc^3+^ and Al^3+^ were also calculated for comparison. This is because they show a stable 3+ state even under a strong reducing atmosphere, unlike Fe, but different dissolution behaviors into CZ or fluorite‐type oxides. Sc with an ionic radius similar to that of Zr can readily dissolve; meanwhile, Al with a smaller radius hardly dissolves in 8‐ or 7‐coordinated sites.

(5) $$\mathrm{Ce}\;\mathrm{sites}:\;Ce_{16}Zr_{16}O_{56}+\frac{1}{2}M_{2}O_{3}+\frac{1}{2}O_{2}\to Ce_{15}M_{1}Zr_{16}O_{56}+{\mathrm{CeO}}_{2}$$

(6) $$\mathrm{Zr}\;\mathrm{sites}:\;Ce_{16}Zr_{16}O_{56}+\frac{1}{2}M_{2}O_{3}+\frac{1}{2}O_{2}\to \;Ce_{16}Zr_{15}M_{1}O_{56}+{\mathrm{ZrO}}_{2}$$

where M = Fe, Sc, or Al. The total energies of Ce_15_M_1_Zr_16_O_56_ and Ce_16_Zr_15_M_1_O_56_ (M = Fe, Sc, and Al) were calculated under the same conditions as those used for cation ordering (Section *3.1.1*). M was doped at all 16 Ce and 16 Zr sites. The total energies of Fe_2_O_3_, Sc_2_O_3_, Al_2_O_3_, CeO_2_, and ZrO_2_ were calculated. **Figure** **3** shows the dissolution energies of Fe^3+^, Sc^3+^, and Al^3+^ in pyrochlore‐Ce_16_Zr_16_O_56_, where the substitution of Ce and Zr sites is plotted as open (red) and filled (blue) circles, respectively. Among the 96 cases (three elements (Fe, Sc, and Al) ×16 positions × 2 sites (Ce and Zr) = 96), one structure that failed to converge (Sc doped into a Zr site), and six structures with outlier total‐energy values were excluded from the plot.

As shown in Figure 3, the median values of the Sc^3+^ dissolution energy are −1.59 and −2.65 eV to the Ce site and Zr site, respectively. The Sc dissolution energy is the lowest among each site, indicating that Sc tends to dissolve at the Ce and Zr sites. This is reasonable because Sc^3+^ readily substitutes Zr^4+^ sites in various oxides.^[^ ^35^, ^36^ ^]^ In the case of Fe, the dissolution energy was also negative for most structures, indicating that Fe^3+^ could dissolve at the Ce/Zr sites. Al dissolution is energetically unfavorable because of the small cation size of Al, particularly for Ce sites with higher ECN values. These results suggest that Fe^3+^ can readily dissolve at the Ce and Zr sites in the CZ. The dissolution energy was also calculated for the Zr‐rich CZ, e.g., Ce_14_Zr_18_O_57_, and shown in Figure S1 (Supporting Information). For the Zr‐rich CZ cases, the same trend but with less difference in dissolution energy depending on dopants was observed, presumably due to more distorted structures than CZ55. Meanwhile, the negative and similar dissolution energies to the Sc‐doping case indicate that Fe seems to be readily dissolved in the Zr‐rich CZ. The relationship between Fe dissolution and low‐temperature cation ordering is discussed in the following high‐temperature in situ XRD analysis.

### 2.2 High‐Temperature In Situ XRD

To clarify the effect of Fe_2_O_3_ on cation ordering in the CZ system, high‐temperature in situ XRD was used to observe the phase change behavior of Fe_2_O_3_‐added Zr‐rich CZ, which was suggested to be more difficult to order by DFT calculations. In high‐temperature in situ XRD, Fe‐containing phases are difficult to identify due to fluorescence when using conventional Cu‐Kα X‐ray. To avoid this problem, a Co‐Kα X‐ray source was used. However, in the case of the 5 vol% Fe_2_O_3_‐added samples, it was still difficult to observe the phase change behaviors. Hence, 50 vol% Fe_2_O_3_‐added Zr‐rich CZ was used to observe clearly Fe‐related phases.

**Figure 4:**
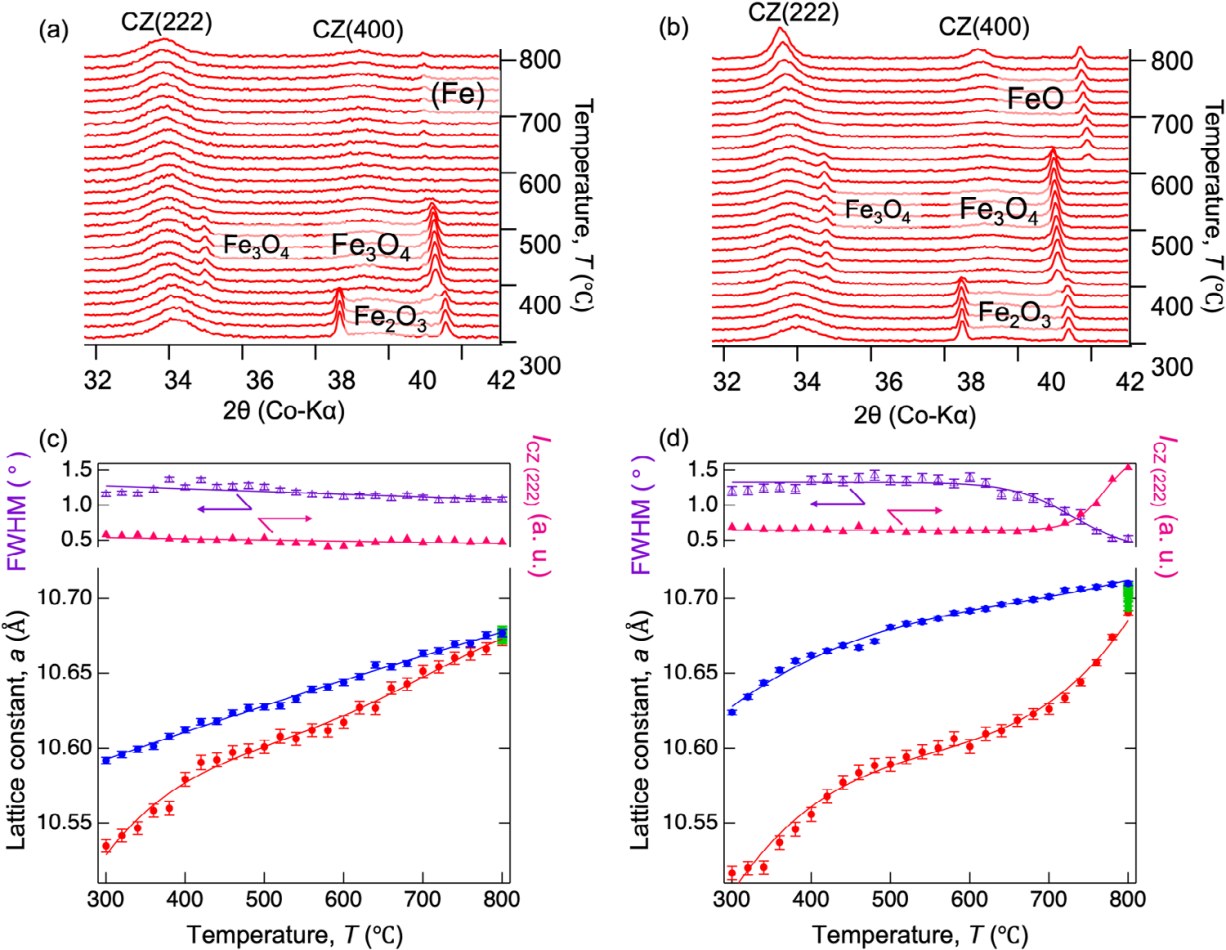
High‐temperature in situ XRD patterns of 50 vol% Fe_2_O_3_‐added Zr‐rich CZ under a) dry 10% H_2_–N_2_, b) wet 4.5% H_2_–N_2_ during the heating process. The analyzed peak intensity of CZ (222), the corresponding full width at half maximum (FWHM), and the lattice constants of CZ are plotted as a function of temperature in c) and d), showing the heating (red), holding at 800 °C for 30 min (green), and cooling (blue) processes. CZ heat‐treated in wet 4.5% H_2_–N_2_ showed significant lattice expansion above 600 °C.

The chemical composition of the Zr‐rich CZ was determined to be Ce_0.434_Zr_0.566_O_2_ by ICP–MS (Table S1, Supporting Information). **Figure** **4**a,b shows the XRD patterns of the Zr‐rich CZs obtained from 300 to 800 °C under dry 10% H_2_–N_2_ and wet 4.5% H_2_–N_2_ gas flows, respectively. As shown in Figure 4a, under a strongly reducing atmosphere (dry 10% H_2_–N_2_), the (222) and (400) peak intensities of Ce‐Zr were almost constant. Fe_2_O_3_ was first reduced to Fe_3_O_4_ at 380 °C and then to Fe at 560 °C without the formation of FeO. In contrast, in a weakly reducing atmosphere of wet 4.5% H_2_–N_2_ (Figure 4b), Fe_2_O_3_ was first reduced to Fe_3_O_4_ at 400 °C, which was then converted to FeO at 640 °C, and the FeO remained stable at 800 °C. In addition, the (222) and (400) peaks of Ce–Zr in the FeO region became sharp and shifted to the lower angle side from ≈760 °C, suggesting that the CZ phase was more deeply reduced in the weakly reducing atmosphere.

To clarify the peak‐sharpening behavior of CZ (222), its intensity and full width at half maximum (FWHM) were plotted as a function of temperature. As shown in the top part of Figure 4c for dry 10% H_2_–N_2_, (d) for wet 4.5% H_2_–N_2_, an increase in the CZ (222) peak intensity and a decrease in FWHM (sharpening) occurred concurrently in the weakly reducing atmosphere ((d) wet 4.5% H_2_–N_2_), suggesting that cation ordering progressed. To confirm the dependence of the reduction behavior on the reducing atmosphere, the temperature‐dependent lattice constant of CZ during temperature increase from 300–800 °C, held at 800 °C for 30 min, and decrease from 800–300 °C is shown at the bottom of Figure 4c,d. The lattice constants were calculated considering the so‐called Z adjustments, that is, height correction. Regardless of the atmosphere, the lattice constant first increased at ≈400 °C, suggesting that, in addition to thermal expansion, Zr‐rich CZ showed chemical expansion behavior due to the reduction between 300 and 400 °C. This indicates that CZ can be partially reduced in this temperature range. Meanwhile, the lattice constant change at 400–600 °C matches the thermal expansion coefficient (TEC) of a typical CZ,^[^ ^37^ ^]^ which is ≈11.8 × 10^−6^. This implies that thermal expansion was observed in this temperature region for both weakly and strongly reducing atmospheres. With a further increase in temperature, a rapid increase in the lattice constant, indicating a deep reduction, was observed only in the case of a weakly reducing atmosphere. This temperature range corresponds to the stable region of FeO and matches the region where peak sharpening occurs, as shown in Figure 4b,d. However, under strong reduction using dry 10% H_2_–N_2_, the change in the lattice constant was not significant (c), suggesting that CZ was not sufficiently reduced. While maintaining the temperature at 800 °C for 30 min (green markers), the lattice constant continued to increase only in a weakly reducing atmosphere (d). Based on these analyses, it was found that, interestingly, CZ order did not occur in a strongly reducing atmosphere in which the added Fe oxide was reduced to metallic Fe, whereas deep reduction led to ordering in a weakly reducing atmosphere in which FeO remained. The deep reduction of CZ due to Fe addition is also supported by other in situ measurement. Figure S2 (Supporting Information) shows the p(O_2_) dependence of the oxygen nonstoichiometry, δ, of Zr‐rich CZ with and without 5 vol% Fe_2_O_3_ by coulometric titration. This indicates that the sample with Fe_2_O_3_ (blue) showed a deeper reduction than that without Fe_2_O_3_ (black). For the sample with 5 vol% Fe_2_O_3_, the deeper reduction starts at log p(O_2_) ≈−16. In addition, the phase boundary of the Fe‐related phases (Fe_3_O_4_, FeO, and Fe) can be clearly observed as a vertical drop because of two‐phase coexistence.

**Figure 5:**
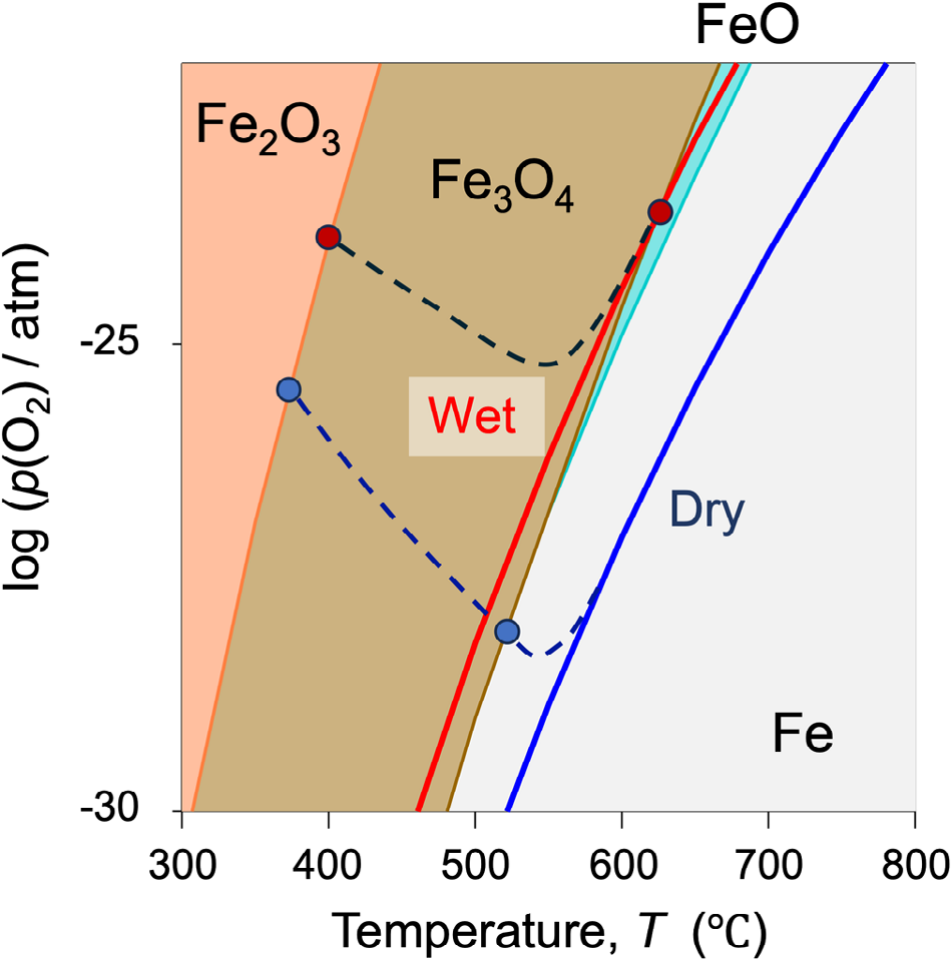
Thermodynamically stable iron oxide phases as functions of oxygen partial pressure and temperature. The bold solid lines indicate the log (p(O_2_)/atm) of wet 4.5% H_2_–N_2_ (red) and dry 10% H_2_–N_2_ (blue) atmospheres. The dashed lines represent the expected trends of log (p(O_2_)/atm) in wet 4.5% H_2_–N_2_ (between red points) and dry 10% H_2_–N_2_ (between blue points), where gas‐solid equilibration is not kinetically fulfilled.

The reduction and cation ordering behaviors were investigated based on the partial oxygen pressure, p(O_2_), in the two gases and the phase equilibrium of iron oxides calculated using FactSage software. **Figure** **5** exhibits the stable phases of iron oxide as a function of p(O_2_) and temperature. It also shows the temperature dependence of log(p(O_2_)/atm) in wet 4.5% H_2_–N_2_ and dry 10% H_2_–N_2_ atmospheres, where the gas compositions were assumed to be 4.5% H_2_–93% N_2_–2.5% H_2_O (wet, red line) and 9.75% H_2_–90% N_2_–0.25% H_2_O (dry, blue line) based on the bubbler temperature and background H_2_O concentration, respectively. The p(O_2_) in the weak reduction (wet) is consistent with the XRD result (Figure 4b), in which the phase transition from Fe_3_O_4_ to FeO occurs at 600 °C, indicating that the phases present are equilibrated at the p(O_2_) above 600 °C. In the strongly reducing condition (dry), the phase transitions from Fe_3_O_4_ to metallic Fe directly without the formation of FeO, also consistent with the high‐temperature in situ XRD patterns, even though the equilibrium boundary (≈500 °C) is slightly different from the XRD result (560 °C in Figure 4a). In the low‐temperature region below 500 °C, significant differences in the equilibrium p(O_2_) and phase boundaries were observed by XRD. The boundary of Fe_2_O_3_/Fe_3_O_4_ (400 and 380 °C for wet and dry conditions, respectively) was not on the curves of the equilibrium p(O_2_), indicating that the gas‐solid phases are far from thermodynamic equilibrium in this temperature region. In other words, the Fe_2_O_3_‐added CZs were reduced via the pathways indicated by the dashed lines, which were provided for visual guidance.

The key finding of this study is that the deep reduction of Fe_2_O_3_‐added Ce–Zr and associated cation ordering cannot be achieved on the blue line in a strongly reducing (dry) atmosphere but only on the red line in a weakly reducing (wet) atmosphere. Iron oxides are not reduced to metallic Fe in a weakly reducing atmosphere; additionally, Fe_3_O_4_ and FeO remain stable over a wide temperature range. Therefore, the precisely controlled p(O_2_), which can sufficiently reduce CZ while keeping Fe as an oxide, is the key factor in the cation ordering of Ce–Zr.

Herein, the reasons for adding Fe_2_O_3_ to induce cation ordering at a low temperature of 800 °C are discussed. First, the effects of other trivalent cation additives, such as Sc_2_O_3_ and Al_2_O_3_, were examined to confirm that adding Fe_2_O_3_ can exclusively enable low‐temperature cation ordering of CZ. Figure S3 (Supporting Information) shows the Raman spectra of 5 vol% Fe_2_O_3_‐added, Sc_2_O_3_‐added, and Al_2_O_3_‐added Zr‐rich CZ after reduction at 800 °C in dry 5% H_2_–Ar. After reduction, a new peak at 450 cm^−1^, which is approaching the ordered 440 cm^−1^ peak, was only observed for the Fe_2_O_3_‐added Zr‐rich CZ, while the peaks of Sc_2_O_3_‐added and Al_2_O_3_‐added CZs remained at 470 cm^−1^. Based on DFT calculations (Section *3.1.2*), Sc_2_O_3_ and Al_2_O_3_ can be fully or partially dissolved in CZ, making them also effective for promoting cation ordering in Zr‐rich CZ. This may be explained by the excellent stability of Sc_2_O_3_ and Al_2_O_3_ with Gibbs free energy, $\Delta G_{f,\;700K}^{\circ }$, of −1700 and −1456 kJ mol^−1^, respectively, which are significantly higher than that of Fe_2_O_3_ (−636 kJ mol^−1^).^[^ ^38^ ^]^ The heat‐treatment temperature of 800 °C was insufficient to form solid solutions with CZ because of their stability. It should also be noted that the degree of cation ordering seems to be limited for the Fe_2_O_3_‐added Zr‐rich CZ, given that the characteristic Raman peak of cation‐ordered CZ (440 cm^−1^) was weak, and a peak at 280 cm^−1^ was not observed (Figure S3, Supporting Information). In addition, the superstructure line at 14.5° in the XRD pattern was difficult to detect after reduction (Figure S4, Supporting Information). Although the superstructure line was confirmed by electron diffraction, as mentioned below, this lower degree of cation ordering agrees with the DFT calculations, which showed a lower energy gain (Table 1).

The dissolution of Fe_2_O_3_ was then analyzed using ICP‐MS for the 5 vol% α‐Fe_2_O_3_‐added Zr‐rich CZ sample, which corresponds to 7.12 mol% of Fe. After reduction, cation ordering, and HCl treatment to remove the excess Fe oxides, the sample contained 7.62 mol% of Fe (Table S1, Supporting Information). In addition, in a previous study, when 5 vol% Fe_2_O_3_ and Co_3_O_4_ were added to CZ55, the transition metals Fe and Co were present at 6.89 and 7.77 mol%, respectively,^[^ ^30^ ^]^ suggesting the full dissolution of 5 vol% Fe_2_O_3_ in CZ.

**Figure 6:**
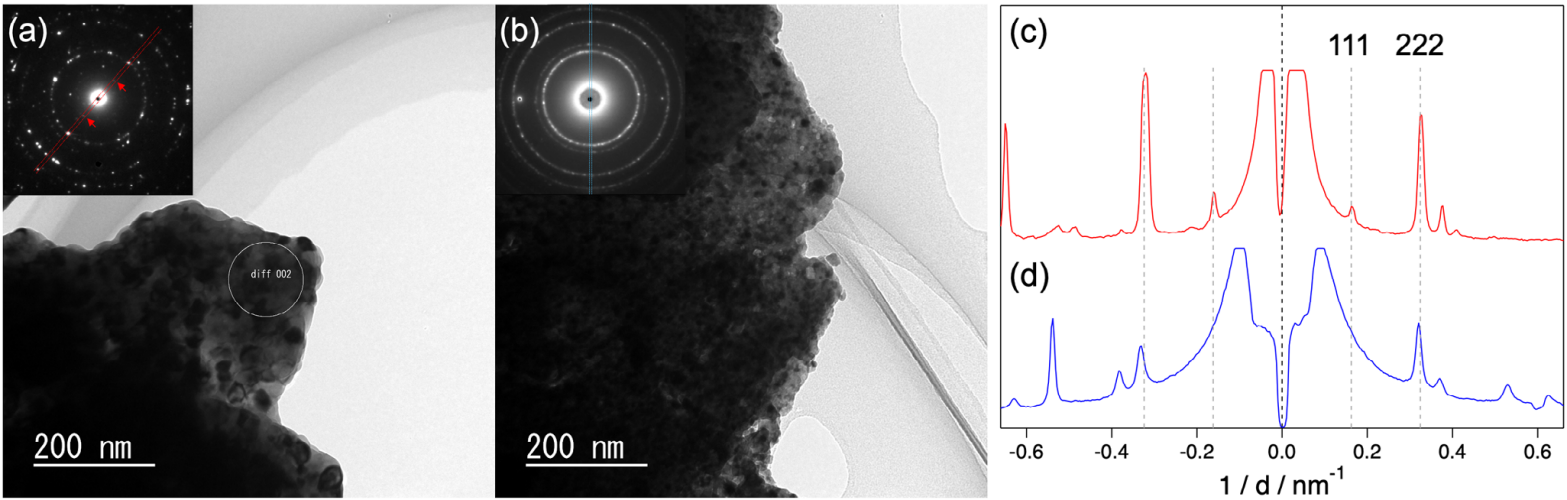
Bright‐field TEM images and electron diffraction patterns of Zr‐rich CZ‐50 vol% Fe_2_O_3_ reduced at 800 °C in a) wet 4.5% H_2_–N_2_ and b) dry 10% H_2_–N_2_ in the high‐temperature in situ XRD measurements. The profiles along the red and blue lines of the diffraction patterns in (a) and (b) are shown in c) and d), respectively. A superstructure peak matching the d‐spacing of CZ (111) was observed only for the sample reduced in 4.5% H_2_–N_2_–wet atmosphere.

To further investigate Fe dissolution and cation ordering, TEM‐EDS analysis was performed for the Fe_2_O_3_‐added Zr‐rich CZ. **Figure** **6** shows the bright‐field TEM images of the Zr‐rich CZ‐50 vol% Fe_2_O_3_ after reduction at 800 °C in a) 4.5% H_2_–N_2_–wet (weak) and b) 10% H_2_–N_2_–dry (strong) atmospheres. These samples were observed after high‐temperature in situ XRD shown in Figure 4. As shown in Figure S5a,b (Supporting Information), a similar relative intensity of the Fe‐Kα peak was observed in the corresponding EDS spectra taken within a 100‐nm‐diameter area in Figure 6a. Furthermore, focusing on the electron diffraction patterns taken in the selected area and inserted in Figure 6a,b, it is evident that the cation‐ordered CZ (a) has a larger grain size, resulting in coarse and disconnected Debye‐Scherrer rings, while the non‐ordered CZ (b) still has fine grains, resulting in uniform and connected Debye‐Scherrer rings. More importantly, superstructure‐derived diffraction spots were observed for cation‐ordered CZ (a) but not for non‐ordered CZ (b). Figure 6c,d show the line profiles of the diffraction patterns in (a) and (b), respectively. Only the sample reduced under a weak reducing atmosphere showed a superlattice peak that exactly matched the d‐spacing of (111). Hence, the cation ordering at 800 °C in Fe‐dissolved Zr‐rich CZ was confirmed by TEM.

**Figure 7:**
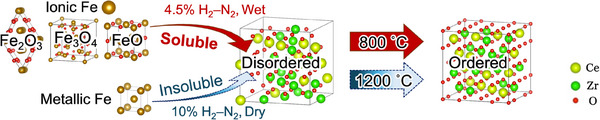
Proposed reaction pathways of Fe_2_O_3_‐added CZ reduced under wet 4.5% H_2_–N_2_ and dry 10% H_2_–N_2_ conditions with respect to cation ordering. The wet 4.5% H_2_–N_2_ atmosphere is essential to keep iron as a cation and to cause the reduction of CZ, simultaneously.

**Figure** **7** depicts the reaction pathways of the Fe_2_O_3_‐added CZ with respect to cation ordering. In a dry reducing atmosphere, disordered CZ is deeply reduced, and Fe_2_O_3_ is converted to metallic Fe, which cannot be dissolved in CZ. In contrast, in a wet reducing atmosphere, Fe_2_O_3_ is reduced not to metallic Fe but to Fe_3_O_4_ with Fe^2+^, Fe^3+^, or FeO with Fe^2+^, which can be dissolved in CZ to facilitate cation migration. To enable these processes, that is the simultaneous dissolution of Fe in CZ under a reducing atmosphere and the deep reduction of CZ itself, precise control of p(O_2_) is essential. It should also be mentioned that more detailed atomistic considerations, such as molecular dynamics simulations, are required to understand how dissolved Fe cations promote cation ordering in future work.^[^ ^39^, ^40^ ^]^

### 2.3 Oxygen Storage Properties and Grain Growth in Zr‐rich CZ

**Figure 8:**
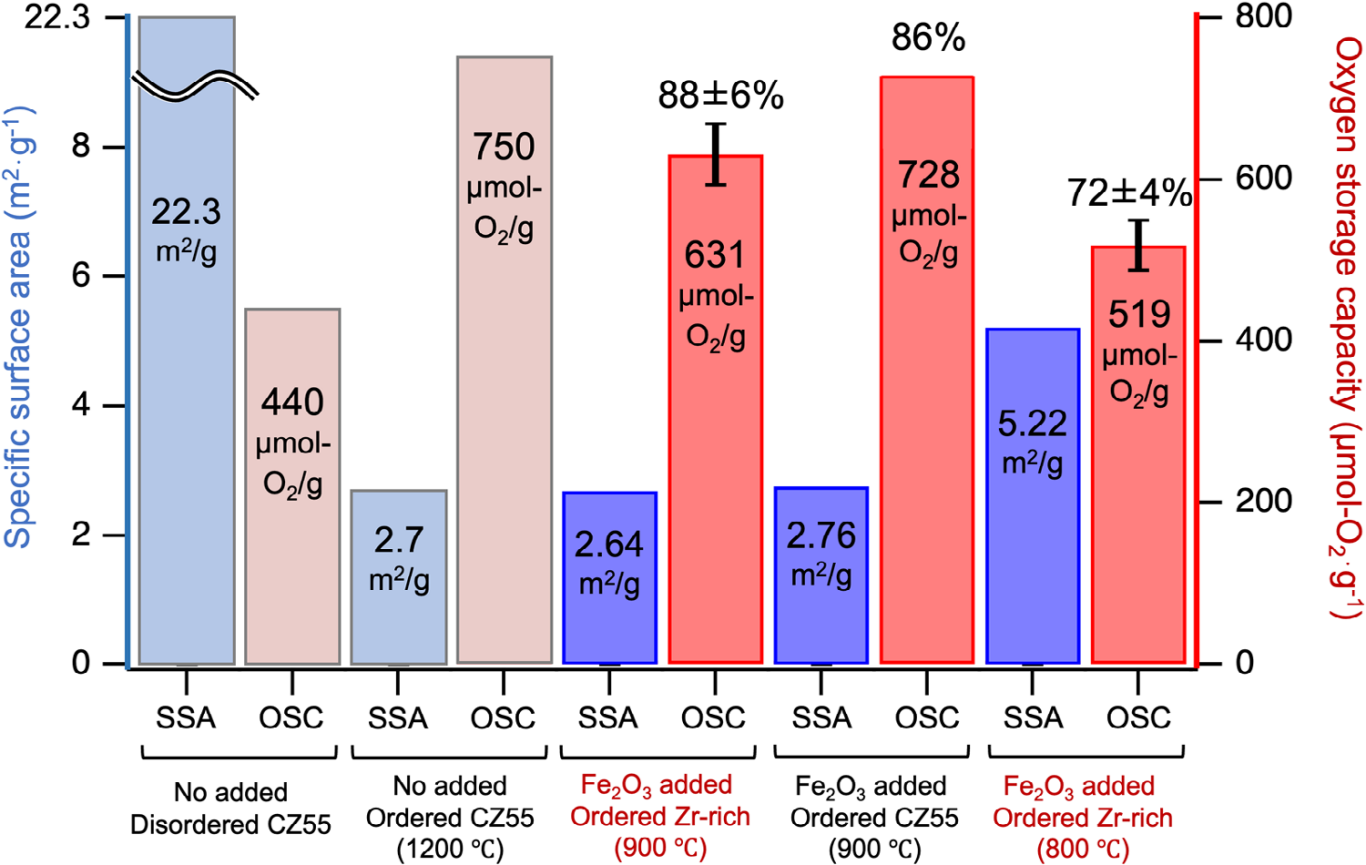
Specific surface area (SSA) and oxygen storage capacity (OSC) at 400 °C for 5 vol% Fe_2_O_3_‐added CZ in comparison with CZ with no Fe_2_O_3_ addition. The temperatures of the reducing treatments are also shown on the sample labels. Fe_2_O_3_ addition led to a decrease in the SSA and an increase in the OSC, even after reduction at 800 °C, suggesting facilitated cation ordering.

In this study, we focused on Zr‐rich CZ, which has good heat resistance and is widely used in practical applications. Low‐temperature cation‐ordered Zr‐rich CZ is expected to exhibit a high OSC, approaching its theoretical capacity. The OSC of 5 vol% Fe_2_O_3_‐added Zr‐rich Ce_0.4475_Zr_0.5525_O_2_, which was reduced in a tube furnace at 900 °C for 3 h under 5% H_2_–Ar for cation ordering, was measured at 400 °C using thermogravimetric analysis (TGA). **Figure** **8** shows the OSCs and specific surface areas of the ordered phases of Fe_2_O_3_‐added Zr‐rich CZ and Fe_2_O_3_‐added CZ55 (Zr/Ce = 1), where disordered CZ55 and ordered CZ55 reduced at 1200 °C are also shown for comparison.^[^ ^26^, ^30^ ^]^ The low‐temperature cation‐ordered Zr‐rich CZ and CZ55 exhibited OSCs of 631 and 728 µmol‐O_2_ g^−1^, which are 88% ± 6% and 86% of their theoretical maximum values, respectively. Thus, Zr‐rich CZ with a high OSC was successfully prepared by adding Fe_2_O_3_ in a weak reduction process.

In addition to the 900 °C sample, the sample reduced at 800 °C is presented in Figure 8, even though cation ordering at 800 °C was limited, as shown in TEM (Figure 6) and Raman spectra (Figure S3, Supporting Information). The SSA and OSC of the 800 °C sample were 5.22 m^2^ g^−1^, which is twice as large as that of the 900 °C sample, and 519 µmol‐O_2_ g^−1^, which is 17.7% lower than that of the 900 °C sample, respectively. This trend, that is, a higher SSA and lower OSC than the 900 °C sample, is reasonable. It should be noted that the OSC of the 800 °C sample was higher than that of the disordered CZ55, even with a smaller SSA by a factor of ≈1/4, owing to cation ordering. Furthermore, 900 °C is still a relatively low cation ordering temperature. These results also suggest that cation ordering and grain growth occur simultaneously, even in the low‐temperature regions of 800 and 900 °C for highly refractory CZ‐based oxides. In this temperature range, cation diffusion in CZ was significantly enhanced, possibly due to the dissolution of Fe ions.

Finally, the impacts of these findings on other applications of CeO_2_ and ZrO_2_, such as solid‐oxide cells, are discussed. In solid‐oxide fuel cells (SOFCs)/solid‐oxide electrolysis cells (SOECs), an intermediate layer is embedded between air‐side electrodes to prevent the formation of a high‐resistivity phase, which causes performance degradation. Sm‐ and Gd‐doped CeO_2_ (SDC and GDC, respectively) with high ionic conductivity and chemical stability are used as the intermediate layer.^[^ ^41^ ^]^ However, a CZ solid solution tends to form between the intermediate layer and the electrolyte at temperatures above 1200 °C, decreasing the electrical conductivity by ≈1 order of magnitude.^[^ ^42^, ^43^, ^44^, ^45^ ^]^ However, this study suggests that Fe dissolution leads to a structural change in the Ce–Zr‐like cation ordering at a low temperature of 800 °C. In addition, Fe‐based oxides are widely used as air‐side electrodes and may react with electrolytes and intermediate layers. Therefore, when the air‐side electrode is exposed to a reducing atmosphere or subjected to severe cathodic polarization during solid oxide fuel cell (SOFC) and solid oxide electrolysis cell (SOEC) operations, Fe diffusion and dissolution from the air‐side electrode may induce the ordering of the CZ at the interface in the SOFC/SOEC, even if they are operated in the desired medium temperature range. Furthermore, because the conductivity of an ordered κ phase at 800 °C is ≈1 order of magnitude higher than that of a disordered t' phase,^[^ ^46^ ^]^ the ordering of CZ in the intermediate temperature range may help prevent a decrease in conductivity between the air‐side electrode and electrolyte. This is also expected to enhance the stability under repeated redox conditions and extend the service life of SOFC/SOEC.

## 3 Conclusion 

Although DFT calculations revealed that Zr‐rich CZ has a smaller energetic gain for cation ordering than stoichiometric CZ55 and is less likely to be ordered, this study successfully achieved low‐temperature cation ordering of Fe_2_O_3_‐added Zr‐rich CZ at 800 °C under a weakly reducing wet 4.5% H_2_–N_2_ atmosphere. High‐temperature in situ XRD was used to identify the phases that cause cation ordering.

CZ ordering occurred in a weakly reducing atmosphere where Fe_2_O_3_ was reduced to FeO, whereas in a strongly reducing atmosphere of dry 10% H_2_–N_2_, Fe_2_O_3_ was reduced to metallic Fe, which cannot contribute to ordering. This suggests that controlling the oxygen partial pressure is important and that a weakly reducing atmosphere promotes the deep reduction of CZ. The ordering behaviors strongly depend on the appropriate chemical stability of the added Fe_2_O_3_ as an oxide and the dissolution energy of Fe ions in the CZ. It is also important to control the reducing atmosphere so that CZ reduction occurs concurrently with Fe dissolution. The OSC of the Zr‐rich CZ was 88% ± 6% of its theoretical capacity at 400 °C. Our study demonstrates that precise control of the reduction atmosphere is effective in promoting ceramic ordering, which is expected to provide valuable insights into a wide range of functional ceramic applications, including SOFCs/SOECs.

## 4 Experimental

Zr‐rich Ce_0.4475_Zr_0.5525_O_2_ and stoichiometric Ce_0.5_Zr_0.5_O_2_ were synthesized by the Pechini method^[^ ^20^, ^47^ ^]^ using Ce(NO_3_)_3_·6H_2_O (99.9%, Kojundo Chemical Laboratory Co.) and ZrO(NO_3_)_2_·2H_2_O (97%, Wako Pure Chemical Ind.).^[^ ^30^, ^48^ ^]^ The molar ratios of metal nitrates, citric acid (99%, Sigma‐Aldrich Co.), and propylene glycol (99%, Wako Pure Chemical Ind.) were 1:3:3 and 1:9:9 for Ce and Zr, respectively. After mixing the metal nitrate, propylene glycol, and citric acid, distilled water (half the weight of the citric acid) was added. The mixture was stirred for 24 h and heated sequentially at 80 °C for 24 h, 150 °C for 12 h, and 300 °C for 12 h. The precursor was then carbonized at 200, 300, and 400 °C for 2 h and calcined at 800 °C for 2 h in air to form an oxide powder. Fe_2_O_3_‐added samples were prepared by introducing α‐Fe_2_O_3_ powder (99.9%, Kojundo Chemical Laboratory Co.) at 5 or 50 vol% of the synthesized CZ powder and mixing them in a planetary ball mill at 300 rpm for 1 h. In addition, during the reduction process in a tube furnace, a uniaxially pressed sample was placed on the same powder inside an Al_2_O_3_ crucible. As shown in Table S1 (Supporting Information), a small amount of Al (≈0.1 mol%) unintentionally introduced as a contaminant from the crucible was detected by inductively coupled plasma mass spectrometry (ICP‐MS).

Powder X‐ray diffraction (XRD; Bruker D8 Advance) was used for phase identification with Cu‐Kα (λ = 1.5418 Å) or Co‐Kα (λ = 1.7903 Å) as the X‐ray source. The lattice constants and crystallite sizes were calculated using the TOPAS4 software. High‐temperature in situ XRD measurements were performed from 300 to 800 °C in an increment of 20 °C. The atmosphere was dry 10% H_2_–N_2_ for a strongly reducing atmosphere or wet 4.5% H_2_–N_2_ for a weakly reducing atmosphere. The wet atmosphere was produced by passing the gas into a water bubbler at room temperature to achieve a partial H_2_O pressure (p(H_2_O)) of ≈2.5%. The OSC was evaluated using thermogravimetric analysis (TGA; TA Instruments Discovery TGA) at 400 °C in three steps: 1) oxidation in synthetic air (60 min), 2) reduction under 5% H_2_–Ar (60 min), and 3) oxidation in synthetic air (60 min). The specific surface area was evaluated by nitrogen adsorption at 77 K and Brunauer–Emmett–Teller (BET) analysis.

Density functional theory (DFT) calculations were performed using the generalized gradient approximation and the Perdew–Burke–Ernzerhof (GGA–PBE) functionals and Vienna Ab initio Simulation Package (VASP) codes.^[^ ^49^ ^]^ Considering that Ce has electrons in its 4f orbitals, the value of U was set to 5 eV based on the literature.^[^ ^50^, ^51^ ^]^ The cutoff energy was 500 eV. The convergence criteria of the self‐consistent field cycles and the ionic relaxation cycles were set to ΔE = 10^−4^ eV and ΔF = 0.05 eV Å^−1^, respectively, and the calculations were performed only at the Γ point.

## Conflict of Interest

The authors declare no conflict of interest.

## Supporting information



Supporting Information

## Data Availability

The data that support the findings of this study are available from the corresponding author upon reasonable request.
