# Supplementary material for: Insights Into Low‐Temperature Cation Ordering in Fe‐Added Ce–Zr‐Based Oxides
Source: Small. 2025 Mar 23;21(16):2412830. doi: 10.1002/smll.202412830 (PMC12019906; doi:10.1002/smll.202412830)
Supplement: Supplementary file 1 — Supporting Information [file SMLL-21-2412830-s001.docx]

Supporting Information

Insights into low-temperature cation ordering in Fe-added Ce-Zr-based oxides

Yume Okazaki, Akihiro Ishii, Itaru Oikawa and Hitoshi Takamura*

Table S1: ICP–MS results for the 5 vol.% α- Fe_2_O_3_-added Zr-rich CZ sample after reduction, cation ordering, and HCl treatment to remove excess Fe oxides.

|  | ^27^Al | ^28^Si | ^56^Fe | ^89^Y | ^90^Zr | ^140^Ce |
| --- | --- | --- | --- | --- | --- | --- |
| Fe_2_O_3_-added Zr-rich CZ  (mol.%) | 0.10 | 1.07 | 7.62 | 0.001 | 51.6 | 39.6 |

Figure S1: Dissolution energies of Fe^3+^, Sc^3+^, and Al^3+^ at the Ce and Zr sites of pyrochlore-type Zr-rich Ce_14_Zr_18_O_57_. The open red and filled blue circles indicate the substitution of Ce and Zr sites, respectively. Zr sites appeared to be energetically favored; however, the difference in the solvation energies was smaller than that in the CZ55 model.

Figure S2: Dependence of oxygen nonstoichiometry on the oxygen partial pressure in 5 vol.% Fe_2_O_3_ added Zr-rich CZ and no-added Zr-rich CZ and differentiation of oxygen nonstoichiometry in 5 vol.% Fe_2_O_3_ added Zr-rich CZ.

Figure S3: Raman spectra of 5 vol.% Fe_2_O_3_-added Zr-rich CZ (ordered), and 5 vol.% Sc_2_O_3_ and 5 vol.% Al_2_O_3_-added Zr-rich CZ after reduction at 800 °C in dry 5% H_2_–Ar. The peaks at 280 and 440 cm^-1^ are related to ordered Ce-Zr, and the peak at 470 cm^-1^ corresponds to disordered Ce–Zr.

Figure S4: XRD pattern after high-temperature *in-situ* XRD at 50 ℃ for 50 vol.% Fe_2_O_3_-added Zr-rich CZ reduced under wet 4.5% H_2_–N_2_. The superstructure line at 14.5° in the XRD pattern was difficult to observe.

Figure S5: EDS spectra of the selected 100-nm region of the Zr-rich CZ-50 vol.% Fe_2_O_3_ after reduction at 800 °C in (a) 4.5% H_2_–N_2_–wet (weak) and (b) 10% H_2_–N_2_-dry (strong). A similar Fe-Ka peak was observed for both samples.
